# Phosphorus-Induced Changes in Microstructure, Optical, and Tribological Behavior of Electrodeposited Ni-P Coatings

**DOI:** 10.3390/ma19091725

**Published:** 2026-04-24

**Authors:** Gabriel Santos, Daniela Santo, Diogo Cavaleiro, Pedro Santos, Sandra Carvalho, Susana Devesa

**Affiliations:** 1 CEMMPRE—Centre for Mechanical Engineering, Materials and Processes, ARISE—Advanced Production and Intelligent Systems, Department of Mechanical Engineering, University of Coimbra, Rua Luís Reis Santos, 3030-788 Coimbra, Portugal; dsanto@dem.uc.pt (D.S.); diogo.cavaleiro@dem.uc.pt (D.C.); sandra.carvalho@dem.uc.pt (S.C.); 2 SRAMPORT Lda., Rua António Sérgio 15, 3025-041 Coimbra, Portugal; psantos@sram.com; 3 Laboratory of Tests, Wear and Materials, IPN-LED & MAT-Instituto Pedro Nunes, Rua Pedro Nunes, 3030-199 Coimbra, Portugal

**Keywords:** nickel-phosphorus coatings, electrodeposition, phosphorus content, microstructure, optical properties, tribological behavior

## Abstract

This work establishes a map between deposition, structure, and properties that enables the design of Ni-P coatings for advanced surface engineering applications. The coatings were electrodeposited on 316L stainless steel substrates using electrolytes of different phosphorus contents, achieved by systematically varying the phosphorous acid (H_3_PO_3_) concentrations. The influence of phosphorus content and intrinsic pH on elemental composition, cathodic current efficiency (CCE), thickness, microstructure, surface topography, crystalline structure, optical properties, and tribological behavior was investigated. The incorporation of phosphorus follows the H_3_PO_3_ concentration increase in a non-linear trend, achieving a maximum value of 22.17 at.% P at the highest bath concentration. The CCE presented an opposite trend, decreasing from approximately 96% to 40%, due to intense activity of hydrogen evolution reactions, and evidencing indirect phosphorus incorporation mechanisms. A transition from crystalline to amorphous structures was observed as the phosphorus content increased, being accompanied by grain refinement and significant roughness reduction to a minimum Sa = 8 ± 1 nm at ~15 at.% P. The optical properties, such as diffuse reflectivity and CIE Lab* color coordinates, were strongly correlated to surface roughness and microstructural evolution, demonstrating the influence of phosphorus through structural changes. Tribological behavior of the coatings revealed a complex interplay between composition, roughness, and wear mechanisms. The lower and more stable coefficients of friction were observed for high phosphorus coatings, although their durability depended on the balance between brittleness and grain refinement. The results demonstrate the combined role of phosphorus concentration and intrinsic pH changes as an effective tool for tailoring the structural, optical, and tribological properties of electrodeposited Ni-P coatings.

## 1. Introduction

The electrodeposition of alloyed nickel-phosphorus coatings has undeniable industrial relevance, materialized by a myriad of distinct applications, which vary according to their properties. These may include tribological, optical, corrosion, or magnetic applications that are heavily imposed by the phosphorus content [1,2,3,4].

In addition, their electrochemical properties are superior, making them prospective electrocatalyst materials for the hydrogen evolution reaction (HER), as well as for applications in ultra-precision manufacturing fields such as the electroforming of X-ray focusing mirrors [5,6,7].

The deposition of such coatings is widely reported, but most of the literature focuses on the electroless variant. However, electrodeposition provides enhanced control over deposition parameters and kinetics. This is particularly valuable for integrated studies that encompass regime transitions and correlations between electrochemical efficiency and structural/functional properties.

On this, the phosphorus content of the alloy serves as a key control parameter that alters the microstructure of the coatings, their topography, wear resistance, and optical properties, such as their reflectivity and lightness. Typically, low-phosphorus coatings exhibit crystalline structures, whereas increasing phosphorus content progressively leads to nanocrystalline and eventually amorphous deposits. The literature places this transition at approximately 16 at.% P [1], although this value represents a range rather than a strict threshold. Moreover, two co-deposition mechanisms have been proposed, and although their underlying phenomena are not consensual, both may certainly occur simultaneously [1].

The direct mechanism of phosphorus incorporation proposes that it occurs via the stepwise reduction of phosphorous acid to elemental phosphorus. This comprises the development of the hypophosphite as an intermediate step and its subsequent reduction, according to Equations (1) and (2), respectively [1,8,9]. Therefore, the formation of stoichiometric Ni-P compounds is typically expected (Equation (3)), as the phosphorus atoms become absorbed and incorporated into the nickel lattice [8].

H_3_PO_3_ + 2H^+^ + 2e^−^ ⇌ H_3_PO_2_ + H_2_O (1)

H_3_PO_2_ + H^+^ + e^−^ ⇌ P + 2H_2_O (2)

*xNi* + *P* → *Nix*P (3)

On the other hand, the indirect mechanism involves the non-Faradaic reduction of phosphorous acid into elemental phosphorus [1,8,9]. In this pathway, adsorbed hydrogen atoms (Equation (4)) facilitate the formation of phosphine (Equation (5)), which acts as an intermediate. The phosphine then undergoes a chemical redox reaction that produces elemental phosphorus (Equation (6)). This sequence may also promote hydrogen evolution reactions at the cathode surface, according to Equation (7).

*H*^+^ + *e*^−^ → *H*_ads_ (4)

*H*_3_*PO*_3_ + 6*H*_ads_ → *PH*_3_ + 3*H*_2_*O* (5)

2*PH*_3_ + 3*Ni*^2+^ → 3*Ni* + 2*P* + 6*H*^+^ (6)

2*H*^+^ + 2*e*^−^ → *H*_2_ (7)

Moreover, these intricate kinetics are influenced by different deposition parameters, such as pH. Its effect is critical, and lower values promote parasitic reactions that decrease the cathodic current efficiency and may induce a change in the prevalent incorporation mechanism [1,6]. The result of such changes has known effects on tribological and optical properties.

The present study assesses the influence of phosphorus content on the microstructure and properties of Ni-P coatings, within the range 0–22 at.% P. This was achieved by varying the content of H_3_PO_3_ in the baths, between 0 and 0.90 mol/L, under constant current density, while considering their intrinsic pH change. The coupling between phosphorus concentration and intrinsic pH makes this system electrochemically complex and is underexplored within the scope of electrodeposited Ni-P coatings.

Therefore, this work aims to systematically investigate the influence of phosphorus content on the electrochemical efficiency, microstructure, surface topography, optical performance, and tribological behavior of electrodeposited Ni-P coatings, establishing integrated structure and property relationships across the crystalline-amorphous transition.

Electrodeposited Ni-P coatings have been widely investigated due to their tunable microstructure and functional properties, particularly across the crystalline-amorphous transition with increasing phosphorus content. Previous studies have demonstrated that low phosphorus contents typically yield crystalline or nanocrystalline structures, while higher contents promote amorphization, significantly affecting hardness, corrosion resistance, and tribological behavior. Additionally, electrolyte composition, pH, and deposition parameters are known to influence phosphorus incorporation and coating properties. In particular, H_3_PO_3_ is commonly used as a phosphorus source, although its simultaneous impact on bath chemistry and deposition conditions introduces additional complexity.

Within this context, several studies have addressed the role of phosphorous acid in Ni-P electrodeposition under different experimental conditions.

Pillai et al. [10] investigated Ni-P deposits with phosphorus contents up to 20 wt.% using direct current electrodeposition from electrolytes containing phosphorous acid as the phosphorus source. The effects of key deposition parameters, including current density, phosphorous acid concentration, phosphoric acid concentration, and temperature, were systematically evaluated in terms of phosphorus incorporation and deposition rate. However, although the authors note that the bath is typically operated at pH values below 1.5 and that phosphoric acid acts as both a buffering and leveling agent, quantitative information regarding the actual pH of the electrolytes is limited. Moreover, pH is not treated as an independent or controlled variable, and its potential coupling with phosphorous acid concentration is not explicitly addressed.

More recently, attention has been given to different deposition modes and their influence on Ni-P coatings. Fukunaga et al. [6] more recently investigated Ni-P electrodeposition from Watts-type baths containing phosphorous acid as the phosphorus source, comparing direct current and various pulse current conditions. In their study, the bath pH was adjusted to fixed values (1.5, 1.3, and 0.9) using sulfuric acid. However, pH was treated as an externally controlled parameter rather than an intrinsic variable of the electrolyte system. As a result, the coupling between phosphorous acid concentration, bath chemistry, and pH evolution during deposition was not explicitly addressed.

Alternative approaches have also been explored to tailor phosphorus content and deposition conditions. Meshram et al. [11] adopted a different approach, developing electrodeposited Ni-P coatings from Watts-based baths containing phosphorous acid as the phosphorus source, but targeting low phosphorus contents (<1 wt.%). In this case, the bath pH was fixed at 2.5, and the phosphorous acid concentration was kept constant. Under these conditions, variations in pH associated with changes in phosphorous acid concentration were not introduced, and therefore their interrelation could not be assessed.

In addition to phosphorous acid-based systems, studies employing alternative phosphorus sources have also been reported. Messaoudi et al. [12] investigated the effect of NaH_2_PO_2_ concentration as a phosphorus source on the properties of Ni-P, Co-P, and Co-Ni-P coatings. In contrast to other studies, a different phosphorus source was employed, and electrodeposition was carried out under potentiostatic conditions. In this work, the bath pH was adjusted to 4 using diluted H_2_SO_4_ and maintained constant throughout the experiments.

Similarly, Benyekken et al. [13] employed potentiostatic conditions and NaH_2_PO_2_ as the phosphorus source, with the bath pH fixed at 2.6. Under these conditions, with constant electrolyte composition and pH not treated as an independent parameter, the experimental design does not provide a basis for examining its influence on deposition behavior and phosphorus incorporation.

Recent studies have also considered the role of additional bath components on Ni-P electrodeposition. Xu et al. [5] evaluated the effects of lactic acid on electrodeposited Ni-P coatings, using H_3_PO_3_ as the source of P, with the bath pH fixed at 4.20. In this study, pH was also adjusted prior to deposition and maintained constant throughout the experiments. Wintachai et al. [14] investigated the microstructural, mechanical, and tribological properties of pulse-electrodeposited Ni-P composite coatings reinforced with monodispersed and bimodal-sized diamond particles. H_3_PO_3_ was used as the phosphorus source, with its concentration kept constant, while the bath pH was maintained at 1.5. For the Ni-P composite coating reinforced with monodispersed 100 nm diamond particles, a coefficient of friction of approximately 0.70–0.75 was observed throughout the steady-state wear period, a value higher than that obtained for the best-performing sample in the present study.

Among the studies considering pH-related effects, Reddah et al. [15] evaluated the influence of H_3_PO_3_-induced pH variation on the electrodeposition behavior, structural development, and functional performance of Ni-P coatings, reporting promising coefficient of friction values. However, the current cathodic efficiency was less favorable, which may limit the process efficiency and increase operational costs during electrodeposition.

Despite extensive studies on electrodeposited Ni-P coatings, the interplay between phosphorus incorporation, electrolyte chemistry, and deposition efficiency remains insufficiently understood. In particular, the role of phosphorous acid (H_3_PO_3_) as both a phosphorus source and a pH-modifying agent introduces coupled effects that are often treated independently in the literature. Ultimately, correlations between microstructure, surface morphology, and combined optical and tribological performance are rarely addressed in a unified framework.

## 2. Materials and Methods

### 2.1. Substrate and Reagents

In this work, quadrangular pieces of 316L stainless steel, 20.0 mm long and 1.00 mm thick, were used as substrates.

The 316L stainless steel is a chromium-nickel austenitic stainless steel with low carbon content. Its typical composition includes 16.0–18.0% Cr, 10.0–14.0% Ni, 2.0–3.0% Mo, ≤2.0% Mn, ≤1.0% Si, ≤0.045% P, ≤0.03% S, and ≤0.03% C, with the balance being Fe [16,17].

This steel is widely used in industries requiring durable, corrosion-resistant materials. It maintains structural integrity under extreme conditions and offers excellent formability and weldability, which explains its attractiveness across diverse applications. Its chemical composition and mechanical properties make it suitable for a variety of uses, from marine environments, due to its high resistance to chlorides, to medical devices, because of its biocompatibility [16,18,19].

The deposition comprised an electrolyte containing nickel(II) sulfate hexahydrate (NiSO_4_·6H_2_O, 98%, Thermo Scientific, Waltham, MA, USA), nickel(II) chloride hexahydrate (NiCl_2_·6H_2_O, 98%, Thermo Scientific), trisodium citrate (C_6_H_5_Na_3_O_7_, 99%, Thermo Scientific), phosphorous acid (H_3_PO_3_, >98%, Thermo Scientific), and boric acid (H_3_BO_3_, ≥99.8%, Fisher Chemical, Pittsburgh, PA, USA). The deionized water was produced in-house using ultra-pure water equipment, and the hydrochloric acid (HCl, 37% *w*/*w*) used for substrate activation was supplied by Panreac (Barcelona, Spain).

In this bath, trisodium citrate stabilizes nickel ions, phosphorous acid provides phosphorus while modifying the pH, and boric acid acts as a buffer to promote uniform deposition.

### 2.2. Electrodeposition Process

The deposition was carried out in a glass container using a two-electrode configuration, with the steel piece as the cathode and a rectangular nickel piece (99.99%, Testbourne Ltd., Basingstoke, UK) as the anode. The electrodes were covered with Kapton tape, leaving a 6 cm^2^ area exposed for deposition.

The bath temperature was maintained at 50 °C, and stirring was performed at 200 rpm using a cylindrical PTFE-coated steel magnetic stir bar (7.60 mm diameter, 45.00 mm length). A current density of 25 mA/cm^2^ was applied for 20 min using an EA-PSI 9360-15 (Elektro-Automatik, Viersen, Germany) power supply. The selected electrodeposition parameters were based on preliminary experimental trials, which identified a stable deposition window yielding homogeneous and adherent Ni-P coatings. Lower deposition times resulted in very small mass gains, increasing the uncertainty of mass measurements, while higher current densities and/or longer deposition times led to a deterioration of coating quality.

Six different baths were tested by varying the phosphorus content through different concentrations of H_3_PO_3_. Initially, the substrate was immersed in an aqueous HCl solution (25% *v*/*v*) for 20 min and rinsed with deionized water. After electrodeposition, the samples were rinsed with deionized water again and dried with a soft tissue.

The mass of each specimen was measured before and after the electrodeposition process using an OHAUS Analytical Plus microbalance (OHAUS Corporation, Parsippany, NJ, USA) with a precision of ±0.00001 g. The resulting mass difference, which corresponds electrodeposited coating, was used to calculate the CCE.

The composition of the electrodeposition baths, along with the intrinsic pH of each bath and the corresponding sample designations, is summarized in Table 1.

### 2.3. Characterization Techniques

The surface morphology of the coatings was analyzed by scanning electron microscopy (SEM) using a Hitachi SU3800 microscope (Hitachi High-Tech Corporation, Tokyo, Japan) equipped with an energy-dispersive spectroscopy (EDS) system (Bruker Nano, Berlin, Germany) for chemical composition analysis at an accelerating voltage of 15 kV. The thickness of the coatings was also obtained through SEM, and its values were estimated from the cross-sectional images.

Surface topography was assessed using atomic force microscopy (AFM), in a Veeco Innova equipment (New York, NY, USA), using tapping mode with a SiN tip (tip radius < 8 nm). AFM images were acquired over an area of 3 × 3 μm^2^, and 2D/3D profiles were generated. The average roughness (Sa) was obtained through the roughness subroutine of the AFM apparatus from three independent measurements.

The crystallographic structure of the coatings deposited on steel substrates was examined by X-ray diffraction (XRD) in grazing incidence mode at 2°, using a Philips X’Pert PRO diffractometer (Malvern Panalytical, Almelo, The Netherlands) with Cu Kα radiation (λ = 1.54060 Å), operating at 45 kV and 40 mA. Measurements were performed over a 2θ range of 10–80°, with a step size of 0.05° and an exposure time of 0.3 s per step.

The reflectivity and color of the coatings were measured using a GretagMacbeth ColorEye^®^ XTH spectrophotometer (X-Rite, Inc., Grand Rapids, MI, USA), which records both specular and diffuse reflection across the wavelength range 360–750 nm and determines color coordinates in the CIE L*a*b* color space, the standard model for human color perception [20,21].

The friction experiments revealed the tribological behavior of the coatings and were conducted on a homemade pin-on-disk tribometer. The tests were carried out under dry sliding conditions at room temperature and a relative humidity of approximately 45–50%. The counter body consisted of a 100Cr6 bearing steel ball and was coupled with a 5 N normal load. During the tests, the coefficient of friction was continuously recorded, and its evolution was interpreted in conjunction with the morphology and elemental composition of the wear tracks obtained by SEM and EDS.

## 3. Results and Discussion

The results are presented in a sequence that reflects the relationships between composition, deposition efficiency, and the resulting properties of the coatings. Firstly, the elemental composition of the coatings is shown (EDS), providing the nickel and phosphorus content, which substantiates the deposition behaviour. Following this, the current efficiency (CCE) is presented, as it quantifies the fraction of charge effectively contributing to deposition. The coating thickness is then reported, being directly related to both the CCE and the composition. Subsequent sections describe surface morphology and topography (SEM and AFM), followed by the crystalline structure (XRD). Finally, optical properties (reflectivity and colour coordinates) and tribological behaviour (coefficient of friction, COF) are discussed.

### 3.1. Elemental Composition

**Figure 1:**
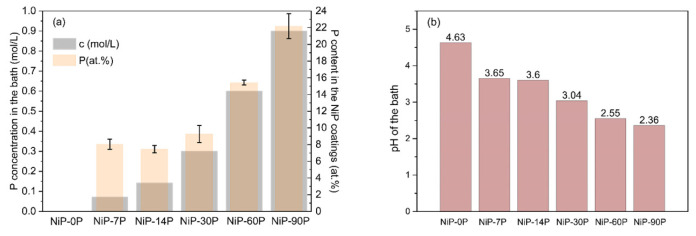
(**a**) Comparison between the phosphorus concentration in the electrodeposition bath (c, mol/L) and the corresponding phosphorus content in Ni-P coatings (at.%), measured by EDS; (**b**) intrinsic pH of the bath at different H_3_PO_3_ concentrations (the pH of the electrodeposition baths was measured using a calibrated pH meter, with readings recorded after full stabilization).

Figure 1a illustrates the relationship between the phosphorus concentration in the electrodeposition bath (c, mol/L) and the incorporated phosphorus content in the Ni-P coatings (P, atomic %). The atomic percentages were obtained from EDS, and the corresponding uncertainties are indicated by the error bars.

The increase in phosphorus concentration in the bath led to a progressive rise in the phosphorus content of the coatings. This relationship is non-linear, as reported by other authors [10], and indicates distinct incorporation regimes.

As expected, no phosphorus is detected in the reference, NiP-0P, confirming that the baseline sample consists of pure nickel. When the bath concentration is raised to 0.07 mol/L (NiP-7P), the phosphorus content in the coating reaches 8.05 at.%, indicating a strong initial sensitivity to phosphorus species in solution. At 0.14 mol/L (NiP-14P), the phosphorus content is 7.45 at.%, which is slightly lower than the previous value but within the error bars also shown in Figure 1a, suggesting that this difference is due to measurement uncertainty rather than a real decrease in incorporation efficiency.

From 0.14 mol/L to 0.30 mol/L (NiP-30P), the phosphorus content rises to 9.28 at.%, maintaining a moderate slope. A significant increase occurs at 0.60 mol/L (NiP-60P), where the phosphorus content reaches 15.42 at.%, and a further increase to 0.90 mol/L (NiP-90P) yields the highest incorporation level of 22.17 at.%. Also, Figure 1b shows a clear trend for pH, as it decreases with an increase in H_3_PO_3_ concentration. These results indicate that, beyond a threshold concentration (~0.3 mol/L), the efficiency of phosphorus co-deposition improves substantially, leading to high-P coatings typically associated with amorphous structures. The pH values, the CCE, and the kinetics of the phosphorus incorporation mechanisms corroborate this. According to Chang et al. [22], both the pH and the H_3_PO_3_ concentration influence the co-deposition process, but the latter becomes considerably relevant for pH values below 3. Therefore, as the H_3_PO_3_ concentration increases, the pH decreases, and their combined effect results in greater phosphorus co-deposition. The observed phenomenon may be attributed to an increased presence of H^+^ ions in more acidic solutions, which favors the indirect mechanism of phosphorus co-deposition [6,23]. This mechanism relies on adsorbed hydrogen (H_ads_) to produce the PH_3_ involved in the non-Faradaic reduction of phosphorus [1]. However, lower pH also promotes the hydrogen evolution reaction (HER), competing for the availability of H_ads_, which would be expected to decrease the co-deposition efficiency of phosphorus [24].

At high concentrations of H_3_PO_3_ (>0.3 mol/L) and intrinsic pH below 3, the co-deposition of phosphorus seems to overcome the competing activity of HER, as will be discussed in Section 3.2. Overall, this trend highlights the complex dependence of phosphorus incorporation on bath composition, governed by electrochemical kinetics and mass transport, while also emphasizing the need to account for experimental uncertainty when interpreting small differences between intermediate concentrations.

### 3.2. Cathodic Current Efficiency

The cathodic current efficiency (CCE) encompasses all electrochemical reactions involving the cathode’s electrons, specifically those contributing to coating deposition (e.g., cathodic reduction of Ni^+^ and the direct mechanism of phosphorus deposition) and parasitic side reactions (e.g., hydrogen evolution), which primarily diminish electron availability and lower efficiency [25].

The CCE was calculated by relating the measured mass change to the applied current and deposition time (Equation (8)), providing an estimate of the fraction of the total charge that resulted in the actual deposition of the coating, rather than in secondary reactions [26]:

(8) $$\mathrm{CCE}=\frac{∆m\times n\times F}{I\times t\times M_{NiP}}$$

where Δm is the mass change in the electrodeposited coating, defined as the change before and after electrodeposition (g); n is the ionic valence of Ni (dimensionless); F is the Faraday constant (C/mol); M is the molar mass of the Ni-P alloy, calculated from the chemical composition measured by EDS (g/mol); I is the applied current (A); and t is the electrodeposition time (s).

**Figure 2:**
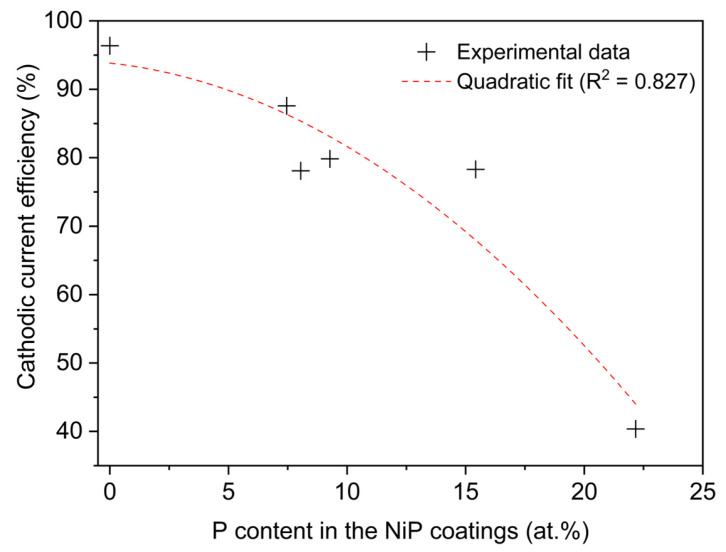
Cathodic current efficiency as a function of phosphorus content (at.%) in Ni-P coatings.

The results presented in Figure 2 reveal a clear correlation between the phosphorus content (at.%) in Ni-P coatings and the cathodic current efficiency (CCE). At 0 at.% P, the efficiency is very high (approximately 96%), but as the P content increases, the efficiency decreases significantly, reaching roughly 40% at 22 at.% P. This trend aligns with theoretical expectations, since higher phosphorus incorporation (>20 at.%) typically occurs under conditions that decrease the CCE [1,6,8], by promoting hydrogen evolution and parasitic reactions [1,3,24]. The decline in CCE is not strictly linear. From 0 to ~10 at.% P, the efficiency decreases moderately (from ~96% to ~80%). However, beyond 10 at.% P, the decrease becomes more pronounced, with values dropping sharply from ~80% to ~40% at 22 at.% P. To capture this non-linear trend, a quadratic regression was applied, yielding a strong correlation (R^2^ = 0.827). This confirms that the relationship between P content and CCE is best represented by a non-linear model, with a more drastic efficiency loss at higher P levels.

Such a decrease in the CCE is widely reported for low-pH baths and is attributed to the intense activity of HER under such conditions [1,3,24]. In acidic solutions, HER comprises three steps [27]: first, the Volmer reaction (electrochemical hydrogen adsorption, H_ads_); then the Heyrovsky and/or Tafel reactions (electrochemical and chemical hydrogen desorption, respectively, H_2_). As HER activity increases, fewer electrons are available for coating reactions, such as the cathodic reduction of Ni^2+^ and the direct mechanism of phosphorus deposition, which becomes evident with a decline in deposition rates [10,28,29].

Nevertheless, under specific conditions, as previously discussed, phosphorus may exhibit greater incorporation at lower current efficiencies. The explanation for this phenomenon may lie in the indirect mechanism of phosphorus incorporation, which involves the chemical reduction of the oxyacid (H_3_PO_3_) to phosphine (PH_3_), and its subsequent reaction with nickel ions to produce elemental phosphorus [1], according to Equations (5) and (6), respectively. Moreover, the formation of phosphine involves the oxidation of adsorbed hydrogen (H_ads_), which is a product of the first step of hydrogen evolution, the Volmer reaction (Equation (4)). Therefore, while hydrogen evolution consumes part of the provided cathode electrons (lowering CCE), it also seems to be a source of H_ads_ for the formation of phosphine and subsequent phosphorus co-deposition [1]. The similar oxidation potentials for phosphine and hydrogen at the electrode surface [9], as well as their competing nature in such environments, corroborate this theory.

Indeed, several authors confirm that the indirect mechanism is prevalent under acidic and low current efficiency conditions, leading to high phosphorus content coatings [6,28,29]. The literature also documents the conjecture linking crystallinity, phosphorus content of the coatings, and the different mechanisms of phosphorus co-deposition [23]. This upholds the existence of two distinct regions: Region 1 where deposition is dominated by nickel reduction, and weak HER activity, leading to higher CCE and the deposition of crystalline coatings with low-to-moderate P content (0–15 at.%); Region 2 that is greatly influenced by hydrogen evolution and chemical (non-Faradaic) phosphorus incorporation, which drastically lowers the CCE, and leads to amorphous coatings with high P content (>15 at.%).

If the complexity of such electrochemical environments is considered, the cathodic reactions certainly involve both mechanisms simultaneously. Under specific conditions, one may be more predominant and potentially explain observed patterns among CCE, phosphorus content, morphology, crystallinity, and topography.

### 3.3. Coating Thickness

**Figure 3:**
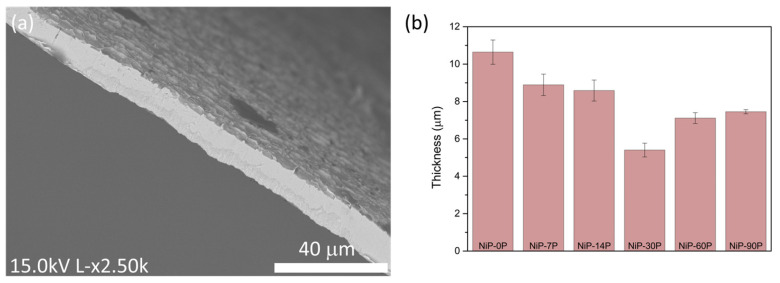
(**a**) Cross-section image of the NiP-0P coating, and (**b**) thickness of the coatings.

The cross-sections of the coatings were analysed using multiple SEM cross-section measurements to ensure statistical representativeness, enabling an estimation of their thickness. Figure 3a presents the cross-section image of NiP-0P, and Figure 3b summarizes estimated thickness values for the whole set of samples. The decrease in thickness is evident with increasing P content until around 15 at.%, ranging from 10.64 μm to 5.40 μm. For incorporation values above this threshold, the thickness of the coatings begins to rise again as more phosphorus is added to the baths, until a maximum of 7.45 μm.

The literature concerning the thickness of Ni-P coatings is limited and consistently regards electroless approaches. Nevertheless, various factors, such as bath temperature, pH, and P concentration, affect the thickness of the deposits. Grizzle et al. [30] conducted a Taguchi statistical analysis to evaluate the quantitative impact of different parameters on the thickness of nickel deposits, estimating an influence of around 41.40% for bath temperature, 35.12% for phosphorus content, 18.24% for deposition time, and 3.87% for surface preparation. The bath pH has also been reported to significantly affect deposition rates and, consequently, thickness [31].

However, these results are not entirely consistent with the literature. An increase in phosphorus is generally associated with a thickness reduction [30,32], and despite it not being fully understood, it may be proposed that the related pH decrease elucidates this correlation. Indeed, lower pH values are responsible for lower deposition rates, and given that the pH value decreased as the H_3_PO_3_ content increased, the samples thickness was expected to decrease across the low to high phosphorus range. Above the 15 at.% threshold, the evolution of coating thickness deviates from the expected trend based solely on pH and cathodic current efficiency, and the explanation may lie in the change in dominant deposition mechanism, where chemical (non-Faradaic) incorporation of phosphorus and autocatalytic nickel ion reduction becomes increasingly significant compared to purely electrochemical deposition, as discussed in the previous section.

Although the addition of phosphorous acid promotes hydrogen evolution and reduces the cathodic current efficiency, this parameter only accounts for the Faradaic contribution to deposition. At higher phosphorus contents, non-Faradaic (chemical) pathways become increasingly significant, including the autocatalytic reduction of nickel ions and the incorporation of phosphorus species. These reactions are not reflected in the current efficiency but contribute to the overall growth of the coating. Furthermore, the formation of phosphorus-rich, more amorphous Ni-P structures enhances the catalytic activity of the surface, facilitating additional chemical deposition. As a result, despite the decrease in electrochemical efficiency, the total deposition rate, and consequently the coating thickness, can increase under high phosphorus conditions.

### 3.4. Surface Morphology and Topography

**Figure 4:**
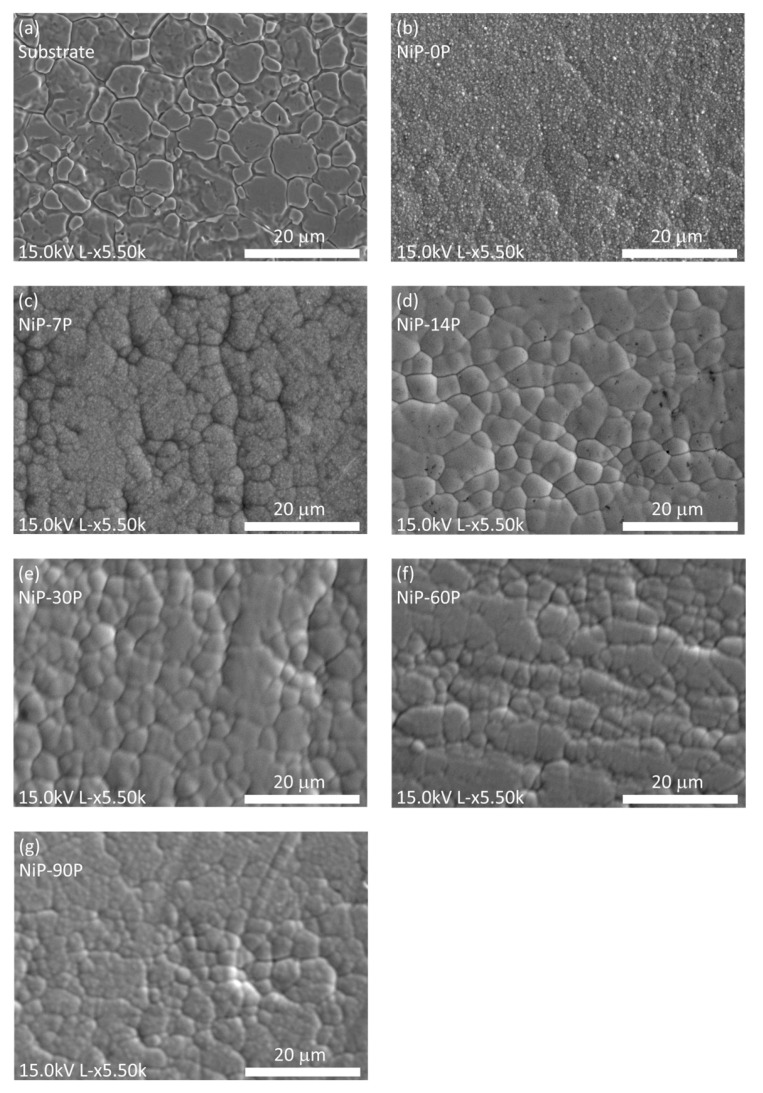
Surface morphology of the (**a**) substrate, and the Ni-P electrodeposited coatings with varying phosphorus content: (**b**) 0 at.% P (NiP-0P); (**c**) ~8.05 at.% P (NiP-7P); (**d**) ~7.45 at.% P (NiP-14P); (**e**) ~9.28 at.% P (NiP-30P); (**f**) ~15.42 at.% P (NiP-30P); and (**g**) ~22.17 at.% P (NiP-90).

Figure 4a shows the surface morphology of the 316L steel substrate. The compact microstructure exhibits characteristic polyhedral surface features with clearly defined boundaries, fissures, and subtle intra-granular texture. Isolated pores may be detected on an overall rough surface.

Figure 4b–g show the surface morphology of Ni-P coatings electrodeposited with different phosphorus contents. The surface morphology exhibits a clear evolution with the introduction of phosphorus and continues to change as its content increases from 0 to 22.17 at.% P.

The coating without phosphorus (0 at.% P, NiP-0P) exhibits a dense surface with typical morphology of crystalline nickel, characterized by spherical-shaped grains.

The incorporation of a small amount of phosphorus (8.05 at.% P, NiP-7P) makes the boundaries of the superficial features more evident and the grains smaller. Further phosphorus incorporation results in smaller, more densely packed features with progressively less-defined boundaries, suggesting the disruptive effect of phosphorus on nickel crystal growth and a tendency toward a typical amorphous or nanocrystalline structure [1,4,33].

This is attributable to the co-deposition of phosphorus in octahedral interstitial sites of face-centered cubic (f.c.c) nickel, which hinders the adsorption of subsequent nickel atoms that take part in crystal growth. Ultimately, as the phosphorus content increases, the disruption of short-range order intensifies, leading to increasingly grain-refined amorphous coatings [1].

Moreover, the morphology of the coatings resembles and appears to follow the substrate morphology to some degree, specifically for low to medium-phosphorus (7.45–9.28 at.%—NiP-7P, NiP-14P, and NiP-30P). Indeed, the nucleation process requires the adsorption of nickel atoms, which subsequently form clusters at energetically favorable sites. Their growth is then inherently influenced by the substrate morphology and by the higher interfacial energy associated with its grain boundaries [25,34,35]. Since increasing H_3_PO_3_ concentration simultaneously decreases bath pH, the observed morphological changes should be interpreted as the combined effect of phosphorus incorporation and electrolyte acidity. Their combined action dictates the grain size and superficial features, the extent of boundaries, and their structure, highlighting the intricacy of such correlations.

**Figure 5:**
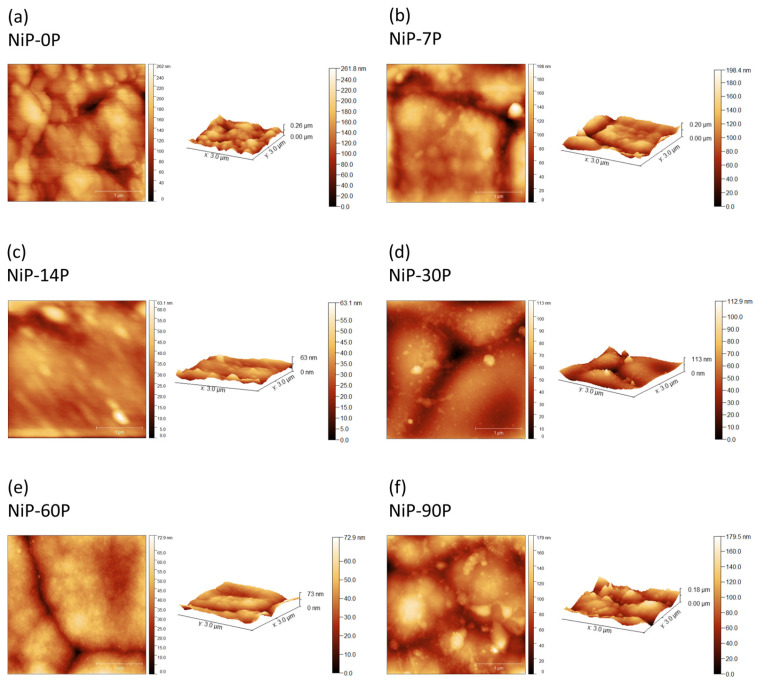
Two-dimensional and 3D AFM images of Ni-P electrodeposited coatings with varying phosphorus content: (**a**) 0 at.% P (NiP-0P); (**b**) ~8.05 at.% P (NiP-7P); (**c**) ~7.45 at.% P (NiP-14P); (**d**) ~9.28 at.% P (NiP-30P); (**e**) ~15.42 at.% P (NiP-60P); and (**f**) ~22.17 at.% P (NiP-90).

**Figure 6:**
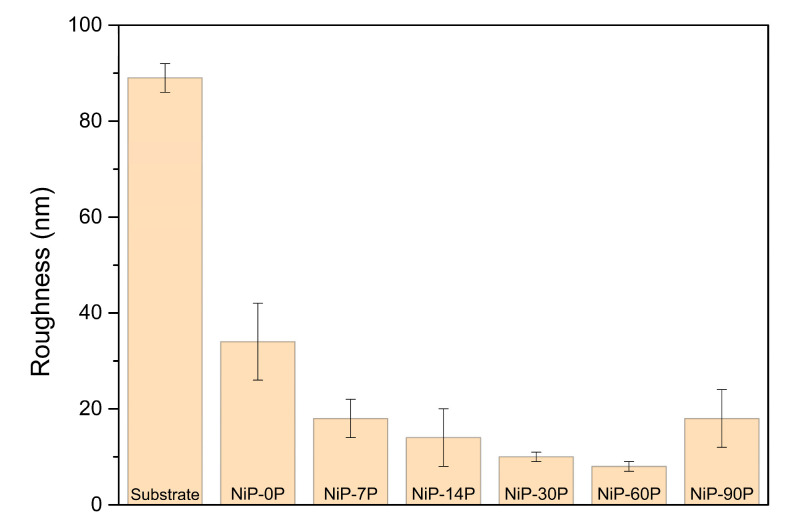
Roughness values (Sa) and respective standard deviation of the substrate and coatings, measured by AFM.

Regarding the topography of the coatings, Figure 5 displays the AFM images of the surfaces acquired over an area of 3 × 3 μm^2^, along with the generated 2D and 3D profiles. The estimated roughness values are summarized in Figure 6.

The roughness of the coatings decreased as the phosphorus content increased (except NiP-90P), with the highest value of Sa = 34 ± 8 (nm) for sample NiP-0P, and the lowest value of Sa = 8 ± 1 (nm) for sample NiP-60P. This trend aligns with the SEM analysis as well as the findings in the literature, which indicate that the phosphorus species in the electrolyte and their co-deposition significantly impact the coatings’ microstructure, and consequently, are responsible for such topography changes [2,36,37]. In this context, the density of nucleation sites, as well as their growth rates and modes, are heavily impacted not only by the phosphorus disruptive effect on the nickel crystalline structures but also by pH variations that are closely coupled to these effects. This is evidenced by the key role of H^+^ in the electrode reactions, which are modulated by pH. Therefore, higher phosphorus incorporation and lower pH values will lead to refined microstructure and preferential horizontal growth rather than vertical, promoting the evening of peaks and valleys of the surface [36,38].

However, the sample with the highest phosphorus content, NiP-90P, marks a shift in this trend, achieving a pronounced increase in surface roughness and its inherent heterogeneity, with Sa = 18 ± 6 (nm). As discussed extensively, the domain of low pH values has several advantageous effects on the coatings’ properties, as well as competing negative ones. The latter includes increasing HER activity, which affects the efficiency of Faradic deposition and consequently the CCE. Effectively, sample NiP-90P exhibits a substantial drop in CCE to ~40%, confirming intense HER activity that may disrupt the growth of nuclei. While bath pH controls the overall tendency for hydrogen evolution, high phosphorus incorporation intensifies local HER activity at the growing Ni-P surface, increasing hydrogen adsorption, which disrupts lateral growth [33,39,40]. Overall, the beneficial influence of phosphorus incorporation initially mitigates the effects of HER, and beyond a threshold, HER becomes the dominant factor governing the deposition process. Other authors have also observed the disruptive effect of HER and associated it with significant surface development at high phosphorus levels (>12 at.%) [40]. This shift accounts for the outlier roughness behaviour and its subsequent impact on the optical and tribological properties.

This corroborates that the combined effect of pH and phosphorus incorporation may be used as a tool for microstructure and topography control, although being limited by heavy HER activity at very low pH values and high phosphorus content. The threshold at which this competing effect shifts is not unanimous, and the transition seems to occur over a range of concentrations, similarly to the amorphization process [1].

### 3.5. Crystalline Structure

**Figure 7:**
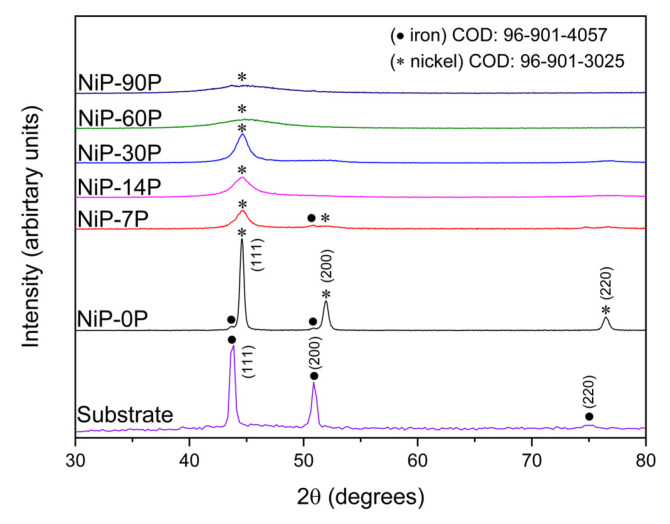
XRD diffractograms of the steel substrate and Ni-P electrodeposited coatings, and the assigned iron (COD ID: 96-901-4057 [41]) and nickel (COD ID: 96-901-3025 [42]) peaks.

Figure 7 shows the XRD diffractograms of the deposited Ni-P coatings, as well as the stainless steel 316L substrate. Characteristic peaks of the 316L stainless steel were observed at 43.9°, 50.9°, and 74.9°, corresponding to the main crystallographic planes of face-centered cubic (f.c.c) iron, respectively (111), (200), and (220) [41]. Indeed, the substrate signals facilitate the identification and analysis of coating-related peaks.

The Ni-P coatings diffractograms show an evident evolution with increasing phosphorus content. The Ni-0P coating exhibits sharp peaks at approximately 44.6°, 51.9°, and 76.5°, corresponding to the (111), (200), and (220) planes of face-centered cubic (f.c.c) nickel, indicating a fully crystalline structure [42]. This is consistent with the EDS results, which, as expected, confirm that the Ni-0P coating is merely composed of nickel. It is also noticeable that the substrate peaks appear in this sample, becoming less visible in subsequent higher phosphorus ones, as the peaks broaden.

For low to medium P content (Ni-7P, Ni-14P, Ni-30P), the (111) nickel peak remains dominant, but peak broadening is observed, suggesting partial amorphization and a reduction in crystallite size. The P content of these and the reported behavior of nickel-phosphorus coatings in the literature correlate well. As phosphorus incorporates into the octahedral interstices of nickel, its crystalline lattice is distorted, reducing long-range order incrementally as phosphorus content increases [1,3,43].

Therefore, the high P coatings (Ni-60P, Ni-90P) exhibit characteristic broadening and a loss of intensity for the preferred texture (111), as well as no signal for the (200) and (220) textures, indicating an overall amorphous structure. The P content levels explain the loss of crystallinity observed in XRD and demonstrate its direct relationship with the crystalline to amorphous transition in Ni-P coatings.

### 3.6. Optical Properties

**Figure 8:**
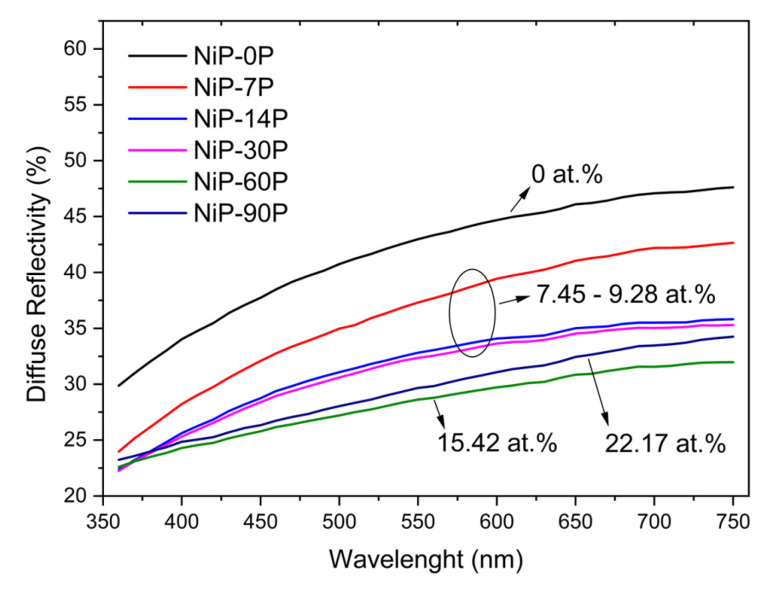
Diffuse reflectivity of the Ni-P coatings across the phosphorus range.

Figure 8 shows the diffuse reflectivity of Ni-P coatings with varying phosphorus content. The evolution of diffuse reflectivity closely follows the changes observed in surface roughness. As the phosphorus content increases, there is an overall decrease in diffuse reflectivity, resulting in potentially shinier surfaces. This behaviour is consistent with the grain refinement induced by phosphorus incorporation, which gradually decreases surface roughness and promotes specular reflection over the diffuse one (scattering) [44]. At high concentrations, a shift occurs in both roughness and diffuse reflectivity. In particular, the coating with 22.17 at.% P (NiP-90P) exhibits a higher diffuse reflectivity than the coating with 15.42 at.% P (NiP-60P), mirroring the corresponding increase in roughness. It is then evident that the diffuse reflectivity is governed to some extent by the roughness of the surface, in agreement with previous studies on Ni-P electrodeposits [45,46].

**Figure 9:**
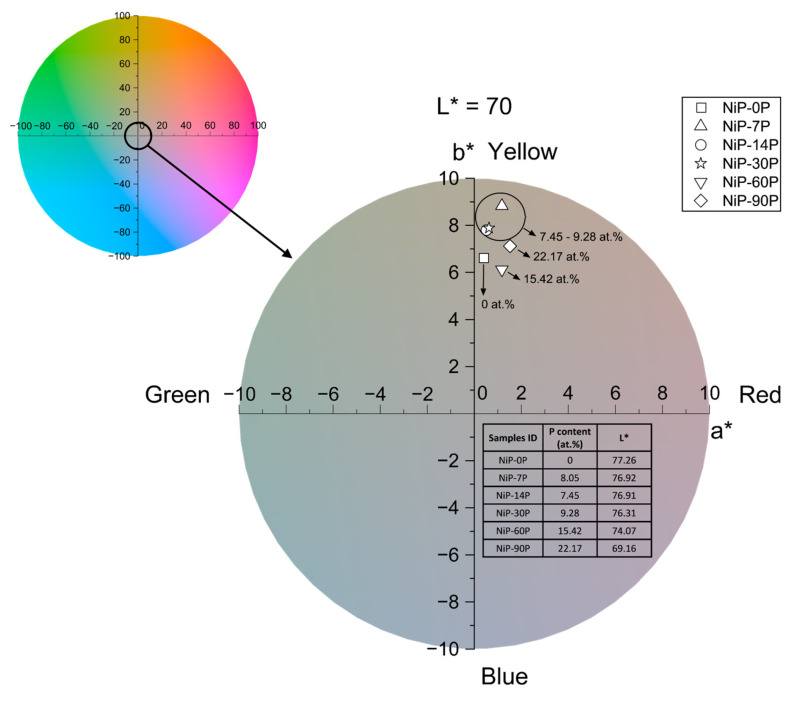
Colour coordinates of the coatings, in the CIE Lab* colour space (inset: lightness, L*, values).

Furthermore, Figure 9 depicts the colour coordinates in the CIE Lab* colour space, comprising lightness (L*), and the chromatic components along the green-red (a*) and the blue-yellow (b*) axes. This is the most commonly used approach for assessing human colour perception, which involves weighting the diffuse reflectivity spectrum according to how the human eye responds to each wavelength [47]. While the specular reflection sustains the fundamentals of shine development on a coating, the diffuse reflection rules the domain of colour. In this context, all samples fall within the yellow-red quadrant (+a*, +b*) with a tendency towards weak yellow hues. As for lightness, L* changes are minimal at low phosphorus content, but an overall decrease is observed, particularly at higher phosphorus levels, resulting in slightly darker coatings. The lowest lightness value observed is L* = 69.16, for sample NiP-90P.

### 3.7. Tribological Behavior

The tribological behaviour of the coatings was assessed considering their coefficient of friction (COF) over 2000 cycles, as well as the morphology of the wear tracks, which allows the identification of wear regimes and failure mechanisms.

**Figure 10:**
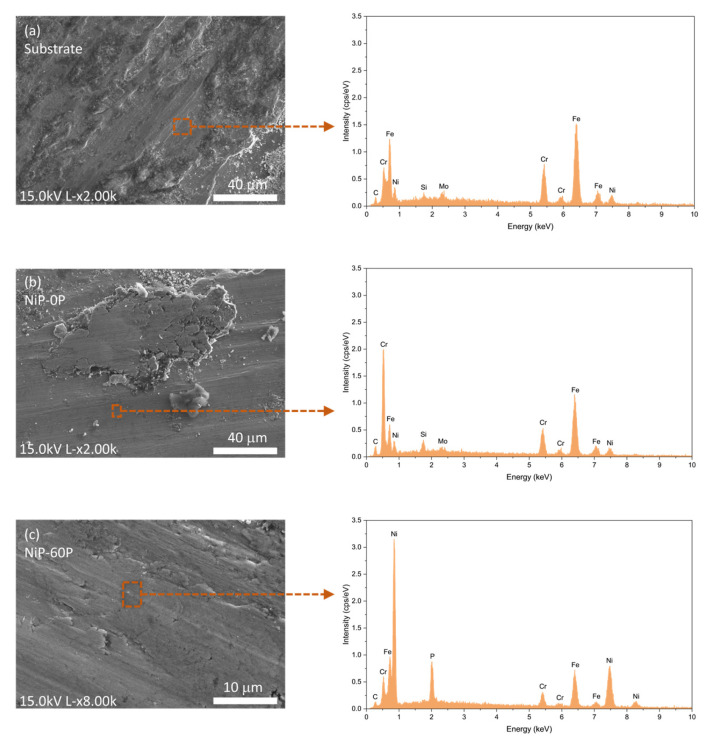
SEM micrographs of the wear tracks of the (**a**) Substrate, (**b**) NiP-0P, and (**c**) NiP-60P, as well as the corresponding EDS spectra.

The EDS analysis provided the elemental composition of the wear tracks, being crucial to verifying whether the coatings withstand the tribological test without reaching the substrate. The consistent presence of phosphorus along the tracks was the deciding factor to determine the presence of the Ni-P coatings, since the 316L substrate contains a significant amount of alloyed nickel that cannot be distinguished from the nickel present in the coatings. Figure 10 shows SEM micrographs and EDS spectra of the substrate, NiP-0P, and NiP-60P. In the phosphorus-containing coatings, such as NiP-60P, the presence of phosphorus along the wear track confirms that part of the coating was still preserved after testing. NiP-14P and NiP-90P likewise showed a consistent presence of phosphorus along the entire wear track. In NiP-0P, which contains no phosphorus, the coating integrity cannot be assessed using phosphorus. Instead, the similarity between the wear track composition and the substrate confirms that the coating was fully removed, exposing the underlying steel.

**Figure 11:**
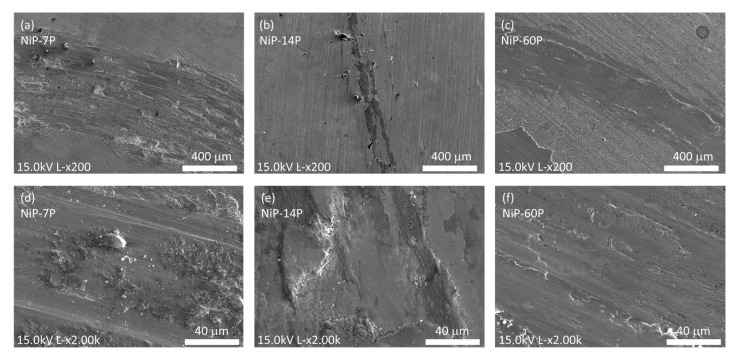
SEM micrographs of the wear tracks of (**a**) NiP-7P, (**b**) NiP-14P, and (**c**) NiP-60P, as well as the respective magnified versions (**d**–**f**).

This interpretation was further supported by the SEM micrographs of the wear tracks displayed in Figure 11. These revealed distinct morphology between the samples where the substrate was exposed (NiP-0P, NiP-7P, and NiP-30P) and those where it remained covered (NiP-14P, NiP-60P, and NiP-90P), as well as the similarity between the substrate and the former ones.

Among samples in which the coating was fully removed, NiP-7P was representative of the observed wear behavior. The wear track exhibited both abrasive wear, supported by the parallel plowing grooves and fine debris, and adhesive wear, manifested through fish-like features caused by plastic deformation [48,49]. This morphology aligned with that of the substrate (Figure 10a), supporting the conclusion that the coating was fully removed and that the observed wear behavior concerns the steel substrate. This behavior directly reflects the wear mechanisms identified from SEM analysis, confirming the transition to substrate-dominated tribological response.

In contrast, the samples retaining part of the coating exhibited two different morphologies. NiP-14P showed a rather subtle wear track, comprising a smooth surface partially covered by a tribofilm of compacted coating wear debris. At higher magnification, adhesive wear was evident, manifested by large wear debris that was plastically deformed by the heavy shear stress. This leads not only to fish-like features but also to microcracking within the wear debris, suggesting the development of fatigue processes under cyclical loading [48]. These mechanisms likely contributed to the formation of loose particles, as observed in Figure 11e. Notably, no significant signs of abrasive wear were detected. The dominance of adhesive wear and tribofilm formation is consistent with the gradual increase in COF observed further, as shear of compacted debris layers progressively raises resistance to sliding.

The wear track of NiP-60P was more evident but remained relatively smooth, with indicative signs of adhesive wear near the edges and large-scale delamination in the surrounding regions. Within the wear track, Figure 11f provides evidence of adhesive debris pullout, microcracking, and localized delamination of lamellar debris, together with subtle plowing grooves indicative of mild abrasive wear. Despite the coexistence of these mechanisms, the limited debris accumulation and smoother contact conditions contribute to the relatively low and stable COF. NiP-90P results were similar to those discussed here.

The wear behavior of Ni-P coatings is governed by a complex interplay of factors, such as composition, thickness, and surface roughness, which do not act independently and often lead to intricate correlations. Nevertheless, phosphorus incorporation is reported to be beneficial at lower levels, enhancing wear resistance, whereas higher phosphorus levels tend to promote brittle behavior due to reduced ductility [1,50,51]. This effect may explain the extensive delamination observed for NiP-60P, as well as for NiP-30P and NiP-90P, which exhibited similar behavior. These delamination events are expected to induce changes in contact conditions, contributing to COF fluctuations during sliding.

Coating thickness is also a critical parameter, as thinner coatings are more susceptible to substrate influence and premature failure [52,53]. However, since all samples exhibited thickness above 5 μm, differences in thickness alone do not appear to explain the observed wear behavior.

As for surface roughness, it affects both the dominant wear mechanisms and the nature of the generated debris, influencing the evolution of the coefficient of friction (COF). In most cases, lower roughness values reduce the number of asperity contacts, thereby limiting debris formation and the development of secondary wear processes [49,54], ultimately lowering the COF values. This trend is consistent with the behavior of NiP-60P, which exhibited the lowest roughness. Nevertheless, the performance of NiP-14P highlights the non-linear nature of these interactions, as its higher roughness appears to be mitigated by the beneficial effect of low phosphorus content on the coating’s microstructure.

**Figure 12:**
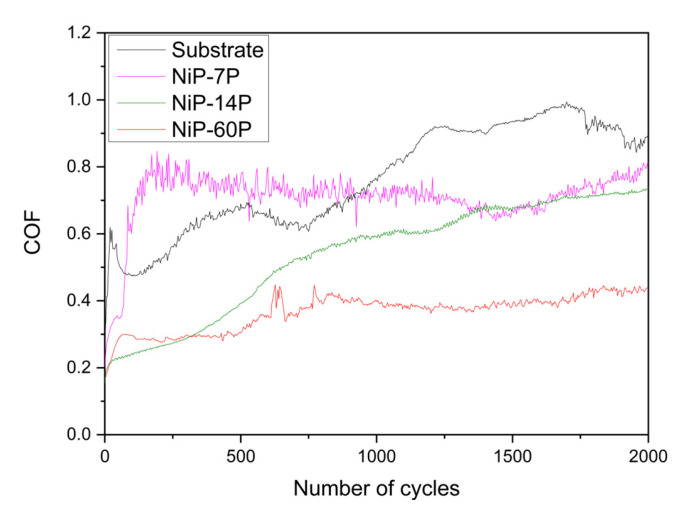
Coefficient of friction of the substrate and Ni-P coatings during 2000 cycles.

The evolution of COF curves, illustrated in Figure 12, underlines the wear mechanisms and coating integrity discussed above. All the samples presented the initial run-in period, during which the contacting surfaces established an equilibrium state, by wearing off higher asperities, superficial oxide films, or forming new ones [55,56,57]. This is followed by a steady-state period that may transition into other states if the roughness, composition, wear mechanism, and debris amount/nature change.

The substrate shows this behavior, with increasingly higher steady-state plateaus ranging from 0.6 to 1.0. Samples in which the coating was fully removed, such as NiP-7P, displayed unstable COF behavior that converged toward that of the substrate, consistent with steel exposure and the incidence of severe abrasive-adhesive wear. In contrast, the samples that retained part of the coating presented distinct trends. NiP-14P shows a gradual increase in COF up to around 0.7, with minor noise throughout the test. This evolution is consistent with the severe adhesive wear observed and the correlated formation of a compacted tribofilm. NiP-60P, on the other hand, exhibited a notably stable COF curve, remaining low over time, with an initial steady-state plateau of around 0.3, which later evolved to approximately 0.4. Its lower roughness and amount of loose debris align well with these lower and more stable COF values, which are typical of high-content phosphorus coatings [58].

Overall, COF fluctuations can be connected to differences in debris formation, delamination events, and surface roughness, reinforcing the microstructural and morphological observations discussed earlier. In addition, although COF is a useful diagnostic signal, wear must be assessed independently, as high phosphorus coatings promote lower values that may not reflect coating durability or integrity alone, as demonstrated here.

## 4. Conclusions

The electrodeposition of Ni-P coatings on 316L steel was achieved successfully within the range of 0–22.17 at.% P. Such a range was carried out by varying the H_3_PO_3_ concentration in the electrolytes. Collectively, these results establish the following main conclusions.

The incorporation of phosphorus became particularly pronounced above ~0.3 mol/L of H_3_PO_3_, with a trend of non-linear increase. This was accompanied by a decrease in pH and the consequent growing influence of hydrogen evolution reactions and the chemical phosphorus incorporation mechanism.

The coatings containing more than ~15 at.% P revealed amorphous structures, evidenced by gradual peak broadening of the XRD diffractograms as the phosphorus content increased. This transition was initiated by the incorporation of phosphorus into nickel interstitial sites, which originates lattice distortion and disruption of long-range order.

The addition of phosphorus induced overall grain refinement and a significant decrease in the roughness of the coatings up to ~15 at.% P. However, the surface growth and heterogeneity were disrupted by intense hydrogen evolution at the highest phosphorus level. Therefore, hydrogen evolution imposes clear restrictions, particularly on how the morphology and topography of a coating can be altered by bath acidity and phosphorus content.

The diffuse reflectivity and CIE Lab* color coordinates were directly correlated with roughness and microstructure changes. Lower diffuse reflectivity was observed for the smoother and refined surfaces, indicating that optical properties are indeed governed by structural modifications induced by phosphorus. This underlines the role of optical characterization as a sensitive and non-destructive indicator of surface and structural changes in Ni-P systems.

The combined effects of phosphorus content, roughness, thickness, and microstructure determined the tribological behavior of the coatings. Lower and more stable coefficients of friction were observed for high phosphorus coatings, due to their smoother surfaces and refined microstructure. Nevertheless, these levels also promoted hydrogen embrittlement and delamination, highlighting the distinction between low friction and coating integrity, as well as the need for combined mechanical and microstructural assessment.

Overall, this work establishes a consistent relationship between deposition, structure, and properties for electrodeposited Ni-P coatings, demonstrating that phosphorus incorporation governs both microstructural evolution and multifunctionality. The study of this compositional window provides practical guidance for tailoring Ni-P coatings for applications requiring controlled reflectivity, low friction, and wear resistance, such as mechanical components, optical finishes, and protective surface engineering solutions.

However, within the defined scope of this study, which focuses on a single phosphorus source, certain limitations remain. This study is limited by the use of a single phosphorus precursor, phosphorous acid (H_3_PO_3_), which restricts a broader understanding of how different P-supplying agents influence phosphorus incorporation and, consequently, the overall properties of the coatings. Future work should therefore include a comparative investigation between H_3_PO_3_ and NaH_2_PO_2_ (sodium hypophosphite) to clarify their respective effects on deposition behavior, microstructure, and resulting coating performance. In addition, further research should address the corrosion behavior of these coatings to better assess their suitability for protective applications.

## Figures and Tables

**Table 1:** Chemical composition of the electrodeposition baths (concentrations in mol·L^−1^), corresponding pH values, and sample identifiers used in this study.

Samples ID	NiSO_4_·6H_2_O	NiCl_2_·6H_2_O	H_3_BO_3_	C_6_H_5_Na_3_O_7_	H_3_PO_3_	pH
NiP-0P	1.00	0.20	0.50	0.50	0.00	4.63
NiP-7P	1.00	0.20	0.50	0.50	0.07	3.65
NiP-14P	1.00	0.20	0.50	0.50	0.14	3.60
NiP-30P	1.00	0.20	0.50	0.50	0.30	3.04
NiP-60P	1.00	0.20	0.50	0.50	0.60	2.55
NiP-90P	1.00	0.20	0.50	0.50	0.90	2.36

## Data Availability

The original contributions presented in this study are included in the article. Further inquiries can be directed to the corresponding authors.
